# Incidental Discovery of Asymptomatic Stage IV Hiatal Hernia With Complete Gastric Thoracic Herniation: A Case Report

**DOI:** 10.7759/cureus.58560

**Published:** 2024-04-18

**Authors:** Murad Qirem, Shahd Yaghi, Byron Okwesili, Raed Atiyat, Yatinder Bains

**Affiliations:** 1 Internal Medicine, Saint Michael's Medical Center, Newark, USA; 2 Gastroenterology and Hepatology, Saint Michael's Medical Center, Newark, USA; 3 Gastroenterology, Saint Michael's Medical Center, Newark, USA

**Keywords:** stage iv, incidental, paraesophageal hernia, asymptomatic, hiatal hernia

## Abstract

Hiatal hernia is a gastrointestinal disorder characterized by abnormal displacement of a portion of the stomach into the thoracic cavity. It has multiple stages ranging from type I-IV according to severity. The more severe the hernia, the more likely it will produce symptoms, and it would be unlikely for it to be asymptomatic. In this case report, we describe a rare situation in which a 79-year-old woman's type IV hiatal hernia was incidentally found after she suffered a mechanical fall.

## Introduction

Hiatal hernia is a gastrointestinal disorder characterized by abnormal displacement of a portion of the stomach into the thoracic cavity through the esophageal hiatus of the diaphragm. It can present in various forms [1].

In hiatal hernia, the usual barrier between the esophagus and the stomach is broken, thus gastroesophageal reflux disease (GERD), which is characterized by heartburn and regurgitation, is a common symptom. Dysphagia, or trouble swallowing, can occur due to displacement of the gastroesophageal junction. Furthermore, some patients may experience chest pain that frequently resembles discomfort related to cardiac issues, which can make diagnosis difficult. There have also been reports of symptoms like bloating, fullness after meals, and belching. [1].

In the following case, we present a type IV hiatal hernia that is entirely asymptomatic and was incidentally found following a mechanical fall. This case represents a rare instance in existing literature because it showcases a complete gastric hiatal hernia within the thoracic cavity without symptomatology.

## Case presentation

**Figure 1:**
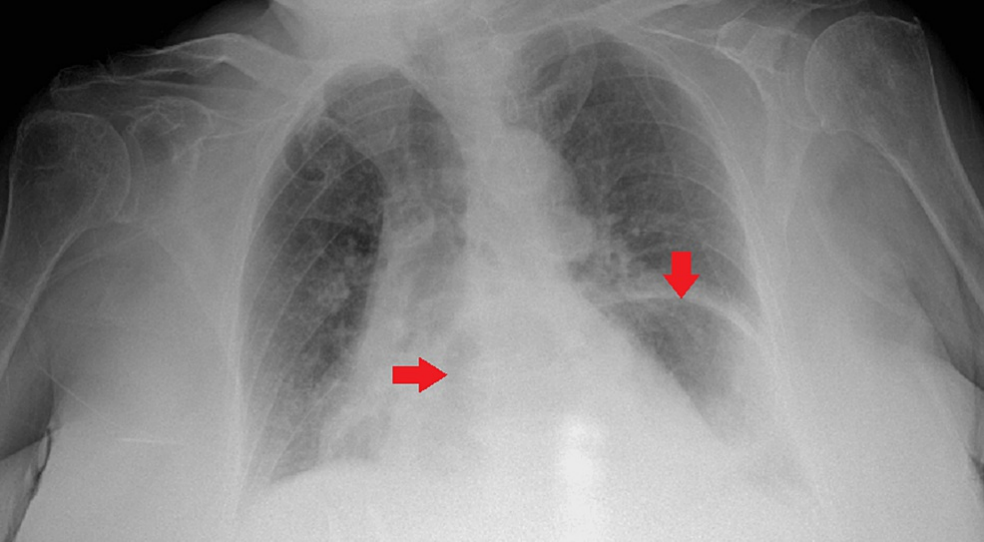
X-ray of a hiatal hernia. Abdominal organs in the thoracic cavity are marked by arrows.

**Figure 2:**
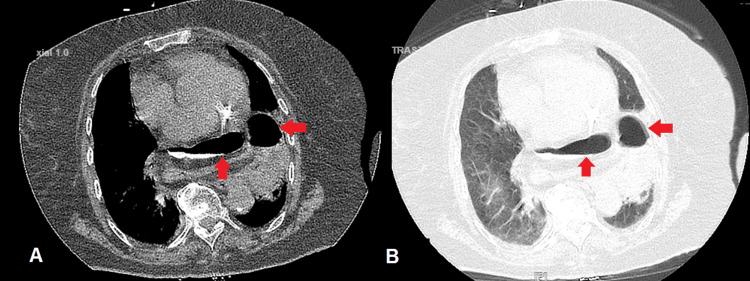
CT chest showing the hiatal hernia. A is the mediastinal window, B is the pulmonary window, and the arrows in both images point to part of the stomach and parts of the colon.

A 79-year-old woman with no significant past medical history presented to the emergency department after a fall while walking to the bathroom in July 2023. She reported that after striking her chest on the ground, she started to feel discomfort in her chest. A chest X-ray was performed to assess for any injuries, revealing findings suggestive of a hiatal hernia, and a region that seemed to indicate consolidation (Figure 1). A CT scan using oral contrast was performed (Figure 2) to get a comprehensive image of the thoracic region after it was determined that additional assessment was necessary.

The CT unveiled a significant diagnosis, a large paraesophageal hernia involving the majority of the stomach and even the colon. A subsequent endoscopy revealed a partial, non-obstructive stomach volvulus without ischemic symptoms and a tortuous esophagus. Surprisingly, despite the severity of the herniation, the patient did not experience any of the symptoms that usually present with such large hiatal hernias such as heartburn, nausea, vomiting, or even frequent belching.

Determining when the paraesophageal hernia formed is more difficult due to the lack of prior chest X-rays or CT scans in the patient's medical history. In this case, the management approach was determined by carefully weighing the risks and benefits of intervention in an asymptomatic patient, taking into account the patient's overall health, age, potential complications related to the hernia, and a comprehensive discussion with the surgical team, it was decided to not undergo surgery, just follow-ups yearly to monitor for symptoms. Her next follow-up appointment will be in July 2024.

## Discussion

Hiatal hernias can be classified into sliding, paraesophageal (rolling), mixed, and complex types of hiatal hernias. A sliding hernia is a type of hernia in which there is displacement of the gastroesophageal junction and part of the stomach in a caudal direction through the esophageal foramen into the thorax [1]. This is the most common type and it results in gastroesophageal reflux disease (GERD) with its related symptoms [2].

On the other hand, paraesophageal hiatal hernia occurs where the stomach herniates into the chest close to the esophagus but does not involve its movement into the chest [3].

The third type is the mixed hiatal hernia, which combines sliding and paraesophageal hiatal hernia features. It involves both the upward sliding of the gastroesophageal junction and the herniation of the stomach beside the esophagus [1].

Lastly is the complex hiatal hernia. This can be the most serious, as herniation may include other organs, such as the colon or spleen. Patients often experience severe symptoms, including chest pain, dysphagia, and respiratory problems, such as shortness of breath, and may require surgical intervention for treatment [4]. In our specific case, the hernia is classified as a complex hiatal hernia, which is surprising because it is completely asymptomatic.

In general, serious complications such as gastric volvulus, incarceration, and strangulation; are more prone to occur in larger hiatal hernias and are life-threatening, requiring surgical intervention. It can also lead to acid reflux, raising the possibility of complications such as Barrett's esophagus and erosive esophagitis. There may also be respiratory symptoms, including dyspnea and cough. This highlights how important early detection and treatment are [5].

Usually, the diagnosis is established based on multiple imaging studies giving an outline of the type and size of the hernia. The most common tests ordered include barium swallow, CT chest and abdomen with oral contrast, endoscopy, and high-resolution esophageal manometry. This will help determine and understand the sub-type of hernia the patient is suffering from for the proper development of treatment procedures while minimizing collateral damages that each sub-type has [1,6].

Most small hiatal hernias do not need surgery, simple lifestyle changes can address the symptoms of acid reflux. Patients are advised not to lie down soon after a meal. This can help prevent reflux and also reduce intra-abdominal pressure. They should also lose weight. Medications such as proton pump inhibitors and antacids are commonly prescribed to alleviate acid reflux and promote esophageal healing. On the other side, patients with severe or complex hiatal hernias can require surgery [7,8].

## Conclusions

In conclusion, there is a wide spectrum of clinical presentations for hiatal hernias, from asymptomatic cases to serious scenarios involving severe complications. There may not always be a direct relationship between the degree of herniation and the intensity of symptoms, as the case mentioned above demonstrates. Enhancing the care and prognosis of patients with hiatal hernias requires a combination of cutting-edge diagnostic techniques, customized treatment programs, and ongoing research initiatives.
